# *In vitro* and field efficacy of fungicides and postharvest 1-methylcyclopropene against dry lenticel rot (*Ramularia mali*)

**DOI:** 10.3389/fpls.2026.1868013

**Published:** 2026-08-03

**Authors:** Stefanie Primisser, Urban Spitaler, Emma Rizzolli, Alex Acler, Werner Rizzolli, Sabine Oettl

**Affiliations:** 1 Institute for Plant Health, Laimburg Research Centre, Auer/Ora, Italy; 2 Department of Agricultural, Forest and Food Sciences (DISAFA), University of Torino, Grugliasco, Italy

**Keywords:** control strategies, latent pathogen, plant fortifier, pome fruits, postharvest rots, psychrotolerant fungi, ripening inhibitor

## Abstract

Dry lenticel rot, caused by the ascomycete *Ramularia mali*, is an emerging postharvest threat to apple production in European alpine regions. Despite its increasing prevalence, established control strategies remain unavailable. This study evaluated the efficacy of 16 fungicides, including succinate dehydrogenase inhibitors (SDHIs; Group 7), demethylation inhibitors (DMIs; Group 3), quinone outside inhibitors (QoIs; Group 11), and multi-site inhibitors, alongside two plant fortifiers. Furthermore, the potential of the ripening inhibitor 1-methylcyclopropene (1-MCP) was tested after harvest. *In vitro* sensitivity assays showed that most tested active substances had limited inhibitory effects, with the exception of metiram and prothioconazole, which achieved 100% growth inhibition at 100 mg L^−1^. Two-year field trials largely reflected these findings, although the plant fortifier acidic clay provided a significant reduction in symptom expression, comparable to the levels achieved by prothioconazole. Notably, application of 1-MCP markedly mitigated disease progression, lowering symptom expression by up to 82–89%. The highest level of control was achieved through the combined application of different fungicides in an integrated strategy followed by 1-MCP application in postharvest. These findings suggest that senescence management may contribute to controlling *R. mali* in commercial apple production. However, the physiological mechanisms underlying the effect of 1-MCP require further investigation.

## 1 Introduction

The ascomycete fungus *Ramularia mali* Videira & Crous has recently been identified as the primary causal agent of dry lenticel rot in apples in northern Italy (Videira et al., 2015; Prencipe et al., 2023; Primisser et al., 2024). This emerging postharvest disease is characterized by the development of small necrotic lesions around the lenticels. Lesions remain superficial and are confined to the peel, with no penetration into the fruit flesh. Symptoms develop during postharvest storage under controlled-atmosphere (CA) or cold-storage conditions, with the earliest occurrence documented after one month (Prencipe et al., 2023; Primisser et al., 2024).

Infected apple fruit remain asymptomatic in the field, which makes it difficult to estimate the true prevalence of the disease. This asymptomatic field phase strongly suggests a preharvest origin of infection, a hypothesis supported by higher recurrence rates in orchards with a documented disease history (Gianetti et al., 2012). Furthermore, a latent asymptomatic phase of the pathogen was hypothesized in a metabarcoding approach by Garello et al. (2023), showing a transition of *R. mali* from an endophytic lifestyle during early fruit development to an epiphytic phase later in the growing season prior to harvest. Recent work has also clarified the taxonomic context of *R. mali* and linked symptom development to additional minor species, such as *R. hydrangeae-macrophyllae* (Primisser et al., 2026a). The economic impact of the disease can be substantial, with losses typically ranging from 10% to 50% depending on microclimate, geography, variety, and storage regime of the affected orchards (Primisser et al., 2026a).

Despite its emergence as a significant threat in the alpine regions of Italy, France, and Austria, information on effective control through available field or postharvest treatments remains limited (Primisser et al., 2026b). In general, fungicide programmes in apple cultivation primarily target major preharvest pathogens, including *Venturia inaequalis*, *Podosphaera leucotricha*, *Colletotrichum* spp., and *Alternaria* spp., as well as fungi that may further affect fruit during storage (Sutton et al., 2014). According to the Fungicide Resistance Action Committee (FRAC), 2025 classification, spraying regimes include diverse active substances, including succinate dehydrogenase inhibitors (SDHIs; Group 7), quinone outside inhibitors (QoIs; Group 11), demethylation inhibitors (DMIs; Group 3), and anilinopyrimidines (Group 9), often used in combination with multi-site inhibitors (Group M04) such as captan. However, although these modes of action are applied against primary pathogens throughout the growing season, they were not specifically established to control latent postharvest pathogens. This may explain why substantial incidences of dry lenticel rot can still be observed after storage of commercially produced fruit. Persistent disease incidence despite intensive fungicide use is particularly concerning in light of other members of the genus *Ramularia*, such as the barley pathogen *R. collo-cygni*, for which widespread resistance or reduced sensitivity to multiple fungicide classes has been well documented (Assinger et al., 2021, 2022; Kiiker et al., 2021; Erreguerena et al., 2022). Whether similar processes occur in this species is unknown, but fungicide baseline sensitivity data are currently lacking.

While *in vitro* assays provide a baseline for the intrinsic toxicity of active substances, they often fail to predict field performance due to the pathogen’s change of lifestyle. A likely transition of *R. mali* from an epiphytic to an endophytic state (Garello et al., 2023) could shield the fungus from contact fungicides, necessitating a comparative study between laboratory sensitivity and multi-year field trials.

Thus, management of *R. mali* faces a dual challenge: the current lack of highly effective fungicidal protocols and the increasing regulatory pressure to reduce fungicide use. This necessitates a shift toward integrated pest management (IPM) strategies that target the pathogen’s ecological requirements. Recent work suggests that the physiological state of the host may be a factor in symptom expression of dry lenticel rot, as *R. mali* degrades cellulose and pectin, but not starch, under storage conditions, leading to the hypothesis that symptom development may accelerate in riper fruit (Primisser et al., 2026a). Thus, delaying senescence may represent a promising strategy to reduce disease progression. The use of 1-methylcyclopropene (1-MCP), a known inhibitor of ethylene-mediated processes (Fan et al., 1999; Watkins et al., 2000; Zhang et al., 2020), may therefore offer a non-fungicidal approach, as its effects on fruit physiology could potentially extend the latent phase of the pathogen.

Therefore, the present study aims to: (i) test the efficacy of 16 commercial fungicides across various chemical classes, as well as two plant fortifiers, through both *in vitro* laboratory assays and two field trials; (ii) determine the first available half-maximal effective concentration (EC_50_) values for two *R. mali* isolates to provide isolate-level reference data for future resistance monitoring and population-level sensitivity assessments; and (iii) investigate the effect of ripening-delay strategies such as 1-MCP treatments on disease incidence, both as a standalone postharvest application and in combination with commonly used field fungicide programmes.

## 2 Materials and methods

### 2.1 Laboratory trials

*In vitro* fungicide tests of commercially available products (**Table 1**) were conducted using two *R. mali* isolates, RM167 (Accession numbers: ITS PP439643, TEF-1α PP480231,RpbII PP480226) and RM023 (Accession numbers: ITS PV982003, TEF-1α PX260106, RpbII PX101873, GAPDH PX244642, ACT PX260013). The fungal isolates were obtained from commercially stored apples (cv. Golden Delicious) from Italy (Province of Bolzano) showing symptoms of dry lenticel rot in 2022. Genetic classification and pathogenicity of the isolates was performed and confirmed as described in Primisser et al., 2026a.

**Table 1:** List of tested fungicides in the *in vitro* assay and field trials: active substance, product name, composition and corresponding FRAC (Fungicide Resistance Action Committee) code are given.

Active substance	Trade name	Manufacturer/Distributor	Composition	FRAC
Acidic clays Bentonite Lignin Sulfonates	Argille Acide	CBC (Europe) S.r.l. Biogard Division	76% 20% 4%	**-** **-** **-**
Boscalid	Cantus^®^	BASF Italia S.p.A.	50 g/100 g	7
Boscalid Pyraclostrobin	Bellis^®^	BASF Italia S.p.A	25.2 g/100 g 12.8 g/100 g	7
Captan	Malvin^®^ 80 WG	UPL Italia S.p.A.	80 g/100 g	M04
Copper	Poltiglia disperss^®^	UPL Italia S.p.A.	20 g/100 g	M01
Dithianon	Delan^®^ 70 WG	BASF Italia S.p.A.	70 g/100 g	M09
Dithianon	Delan^®^ Pro	BASF Italia S.p.A.	9.10 g/100 g	M09
Potassium-phosphonate			40.87 g/100 g	P07
Dodine	Syllit^®^ 65 Syllit^®^ 544 SC	UPL Italia S.p.A.	65 g/100 g 355 g L^−1^	U12
Fludioxonil	Geoxe^®^	Syngenta Italia S.p.A.	50 g/100 g	12
Fluxapyroxad	Sercadis^®^ SC	BASF Italia S.p.A.	26.5 g/100 g	7
Mefentrifluconazole	Revysion^®^	BASF Italia S.p.A.	75 g L^−1^	3
Metiram*	Polyram^®^ DF*	BASF Italia S.p.A.	70 g/100 g	M03
Penthiopyrad	Fontelis^®^	Corteva Agriscience Italia S.r.l.	20.4 g/100 g	7
Prothioconazole**	SIP41061 **	Sipcam Oxon S.p.A.	400 g L^−1^	3
Potassium-phosphonate	Century^®^ Pro	BASF Italia S.p.A.	51.7 g/100 g	P07
Pyrimethanil	Scala^®^	BASF Italia S.p.A.	36.7 g/100 g	9
Pyrimethanil Fludioxonil	Pomax SC	Certis Belchim B.V.	336 g L^−1^ 133 g L^−1^	9 12
Sulfur	Tiovit^®^ Jet	Syngenta Italia S.p.A.	80 g/100 g	M02
Trifloxystrobin	Flint^®^	Bayer CropScience S.r.l.	500 g L^−1^	11

Fungicides for baseline treatments against apple scab in the field trials are excluded from the list.

* Authorization for Metiram in European Union expired in November 2024.

**Not labelled for apple in European Union

Fungicide-amended media were prepared by dissolving commercial products (**Table 1**) in sterile deionized water to create a 1,000 mg L^-1^ active substance stock solution. This stock was serially diluted and added to potato dextrose agar (PDA; Liofilchem, Italy; 42 g L^-1^) cooled to 45 °C to achieve final concentrations of 100, 10, 1, 0.1, and 0.01 mg L^-1^. To prevent bacterial contamination, streptomycin sulfate (50 mg L^-1^; ≥ 98%, Fluka, Honeywell, Germany) was added to the medium post-autoclaving at the same temperature. The mixture was poured into 90 mm Petri dishes, which were subsequently sealed with parafilm to prevent desiccation.

To prepare the mycelia for the fungicide assay, a mycelial plug from the −80 °C glycerol stock was crushed in a mortar with 3 mL of deionized sterile water. An aliquot of 800 µL was transferred onto a PDA plate, spread with a Drigalsky spatula, and incubated for one week at 20 °C. From the freshly grown fungus, fungicide sensitivity was assessed by transferring 1 × 1 mm mycelial plugs upside down onto the fungicide-amended and control plates, so that the mycelial side was directly placed in contact with the active substance. Four replicate plates were prepared for each isolate, active substance (AS), and fungicide concentration. The plates were incubated in the dark at 20 °C for three weeks. The increase in mycelium for this slow-growing species was determined by measuring the horizontal and vertical diameters weekly, using the mean of both measurements with adjustment by subtraction of the inoculation plug. The weekly growth rate (WGR) of the mycelium was calculated as a linear increase in diameter using the formula:


$$WGR=\left(D_{W3}-D_{W1}\right)/2$$

where *D* represents the measured mycelial diameter in mm, and *W3* and *W1* indicate week three and week one, respectively.

Inhibition of mycelial growth was calculated by comparing the WGR on fungicide-amended agar to the WGR on control agar without fungicide. For each active substance (AS), percent inhibition was determined based on the corresponding WGR values.


$$\text{Inhibition }\left(\%\right)\;=\;\left[1\;-\;\left(WGR_{treatment}/WGR_{control}\right)\right]\;\times \;100$$

### 2.2 Field trials

The same apple orchard was used for both field trials in 2022 and 2023. The orchard was managed according to the AGRIOS 2021 guidelines for integrated pome fruit production, harvesting, storage, and marketing of apples in South Tyrol (northern Italy) (AGRIOS; www.agrios.it). The orchard was protected by a hail net, the cultivar Golden Delicious was grafted onto M9 rootstock and trained as a slender spindle with an average height of approximately 3.5 m. Each experimental plot contained 15 consecutive trees in a single row. The first three and the last three trees of a plot bordering the neighbouring plot were not considered for apple sampling. The experiments were set up in a randomized block design with four replicates, where a row constituted a block. Between each block, two untreated rows prevented a potential influence of spray drift.

To determine the appropriate harvest window for the growing area, ripening indices were assessed in both experimental years one day prior to harvest. Apples were harvested on 13 September 2022 and 12 September 2023. Starch index, flesh firmness, soluble solids content (SSC), and titratable acidity (TA) were measured one day before harvest on a representative fruit sample collected from the orchard. Starch degradation was scored using a 1–10 iodine-staining index (Ctifl scale). Flesh firmness, SSC, and TA were determined using an automated fruit quality analyzer (Pimprenelle, Setop Giraud Technologies, Cavaillon, France), with firmness expressed in kg cm^−2^, SSC expressed in °Brix, and TA expressed as g malic acid equivalents L^−1^. Mean values (± SD) in 2022 were 3.04 ± 0.26 for the starch index, 6.89 ± 0.35 kg cm^−2^ for flesh firmness, 11.33 ± 0.47°Brix for SSC, and 4.96 ± 0.44 g L^−1^ malic acid for TA. In 2023, the corresponding values were 3.01 ± 0.20, 6.71 ± 0.15 kg cm^−2^, 10.51 ± 0.59°Brix, and 4.19 ± 0.36 g L^−1^ malic acid, respectively. These ripening indices confirmed that the fruit was harvested within the commercial maturity window established for the growing region. Weather conditions during the two field-trial years, 2022 and 2023, are summarized below. In 2022, temperatures ranged from −8.0 °C to 38.1 °C, with an annual average of 12.9 °C, and total precipitation amounted to 608.2 mm across 93 rain days, with the highest monthly rainfall recorded in June (117.5 mm) and lower precipitation in July and August (52.7 and 36.6 mm, respectively). In 2023, total precipitation reached 901.4 mm over 115 rain days, with high rainfall recorded in July (164.1 mm) and October (173.0 mm), while average temperatures remained comparable to 2022 at 13.1 °C, with a range of −7.1 °C to 36.9 °C. In September, rainfall totalled 80.3 mm over 10 rain days in 2022 and 23.7 mm over five rain days in 2023. Meteorological data were recorded by a weather station located in the same district as the experimental orchards. The complete monthly data are publicly available at https://meteo.laimburg.it.

### 2.3 Field treatments

In both years, boscalid (0.25 g L^−1^ Cantus^®^), dithianon and potassium phosphonate (2.5 mL L^−1^ Delan^®^ Pro), dodine (0.9 g L^−1^ Syllit^®^ 65; 0.8 mL L^−1^ Syllit^®^ 544 SC), fludioxonil (0.3 g L^−1^ Geoxe^®^), mefentrifluconazole (1.3 mL L^−1^ Revysion^®^), pyrimethanil (1 mL L^−1^ Scala^®^), and a fungicide strategy were tested. Additionally, a treated control and an untreated control were included. In 2023, two treatments were additionally tested: prothioconazole (0.3 mL L^−1^ SIP41061) and acidic clay (15 g L^−1^ Argille Acide). In 2022, test products were applied four times on 30 June, 19 July, 23 August, and 6 September. The fungicide strategy included four applications of pyrimethanil and fludioxonil, boscalid, pyraclostrobin, and dithianon with potassium phosphonate. For dodine, the first two applications were carried out using Syllit^®^ 65, whereas subsequent applications were performed with Syllit^®^ 544 SC.

In 2023, the applications of the test products were applied six times on June 19, July 5, July 17, August 2, August 18, and August 30. The fungicide strategy included five applications of dodine and a final application of fludioxonil.

Additional fungicide treatments, which were applied to avoid any impact by other pathogens such as *Venturia inaequalis* and *Podosphaera leucotricha*, were applied in 2022 on 5 July, 2 August, and 23 August and consisted of captan (Malvin 80 WG, 800 g kg^−1^, UPL Italia S.p.A.) at a dosage of 2 kg product per hectare and tetraconazole (Domark 125, 125 g L^−1^, Gowan Italia S.r.l.) at a dosage of 250 mL product per hectare. In 2023, these treatments were applied five times on 26 June, 17 July, 28 July, 18 August, and 30 August with captan (Merpan 80 WDG, 800 g kg^−1^, ADAMA Italia S.r.l.) and tetraconazole (Lidal, 40 g L^−1^, Corteva Agriscience Italia S.r.l.).

All treatments were applied using a plot sprayer (Typ Q/014; Waibl Sprayers, Italy) with four tanks of 200 L capacity, an axial fan, and a fan attachment. The spray rate was 1,000 L per hectare. Application was performed at a driving speed of 5.0 km h^−1^ using eight injector nozzles (Albuz CVI 80 03) on each side of the sprayer at 13.5 bar. The treatments were applied from both sides of the row. The untreated control received no fungicide applications. The treated control received the standard background treatments required for general orchard management, but no targeted treatments against *Ramularia*. Insecticides, fertilizers, and all further agronomic measures were applied uniformly to the whole orchard.

### 2.4 Ripening inhibitors and storage

Treatment with 1-methylcyclopropene (1-MCP; SmartFresh™, AgroFresh, Philadelphia, PA, USA) was performed exogenously after six days of cold storage acclimation. The SmartFresh™ formulation contained 0.14% 1-MCP, and was applied at a final concentration of 1 μL L^−1^ (equivalent to 1.6 g·m^−3^), in accordance with the manufacturer’s instructions. The treatment was conducted in an airtight container maintained at 1.3 °C for 24 h. Upon completion of the exposure period, the fruit were moved to commercially used controlled-atmosphere storage cells for 8 months. Apples without 1-MCP treatment were stored for the same duration as the treated variants.

### 2.5 Disease assessment after storage

Stored apples were divided into infection classes to assess disease severity: no symptoms (infection index 0), less than 1 cm² symptomatic fruit surface (infection index 1), 1–4 cm² symptomatic fruit surface (infection index 3.5), 4–9 cm² symptomatic fruit surface (infection index 7.5), and more than 9 cm² symptomatic fruit surface (infection index 9). For the 2022 trial, disease assessment was performed on 5 July 2023. For the 2023 trial, disease assessment was performed on 30 May 2024.

### 2.6 Statistical analyses

All statistical analyses were conducted in R version 4.4.3 using RStudio 2025.05.0.

Inhibition data were analyzed using a zero-inflated beta mixed-effects model implemented in the glmmTMB package (McGillycuddy et al., 2025). The model included isolate, active substance (AS), and concentration (conc) as fixed effects, with an interaction term between AS and concentration (AS × conc). Replicate identity was included as a random intercept. Zero inflation was modeled as a function of isolate. To meet the assumptions of the beta distribution, values equal to 1 were adjusted to 1 − ϵ (ϵ = 1 × 10^−6^). Estimated marginal means were computed using the emmeans package (Lenth and Piaskowski, 2026) to evaluate differences among active substances at a concentration of 100 mg L^−1^. Pairwise comparisons were performed with Tukey adjustment for multiple testing.

Dose–response relationships between fungicide concentration and mycelial growth inhibition were analyzed for the active substances which showed 100% inhibition.

For each isolate–fungicide combination, a four-parameter log-logistic model (LL.4) was fitted using the drc package (Ritz et al., 2015), with the lower asymptote constrained to zero and the upper asymptote estimated from the data. From each successfully converged model, the median effective concentration (EC_50_) and its corresponding 95% confidence interval were extracted.

Field efficacy of fungicides was analyzed using linear mixed-effects models implemented in the lme4 package (Bates et al., 2015).

The size of *Ramularia*-infected area on fruit surface was modelled as a function of treatment as a fixed effect, while sampling position (upper and lower canopy) was included as a random intercept. Models were fitted using maximum likelihood estimation. Treatment effects were assessed by analysis of variance (ANOVA) on the fitted model.

1-MCP efficacy data were analyzed using a linear model, with treatment as a fixed factor. The model was fitted using ordinary least squares estimation. Treatment effects were evaluated by ANOVA.

Pairwise comparisons among treatments were performed using Tukey’s honestly significant difference (HSD) test with adjustment for multiple comparisons, implemented via the multcomp package (Hothorn et al., 2008). Statistical significance was assessed at *P* ≤ 0.05.

Graphical results were shown by using the packages ggplot2 (Wickham, 2016).

## 3 Results

### 3.1 Laboratory trials

A mixed-effects zero-inflated beta regression showed no significant effect of isolate on inhibition (likelihood-ratio test: χ²(1) = 0.002, P = 0.97). Therefore, data from both isolates were pooled for subsequent analyses.

For the general inhibition analysis, data from both isolates were pooled because no significant isolate effect was detected. Dose–response models for metiram and prothioconazole were fitted separately for each isolate.

**Figure 1:**
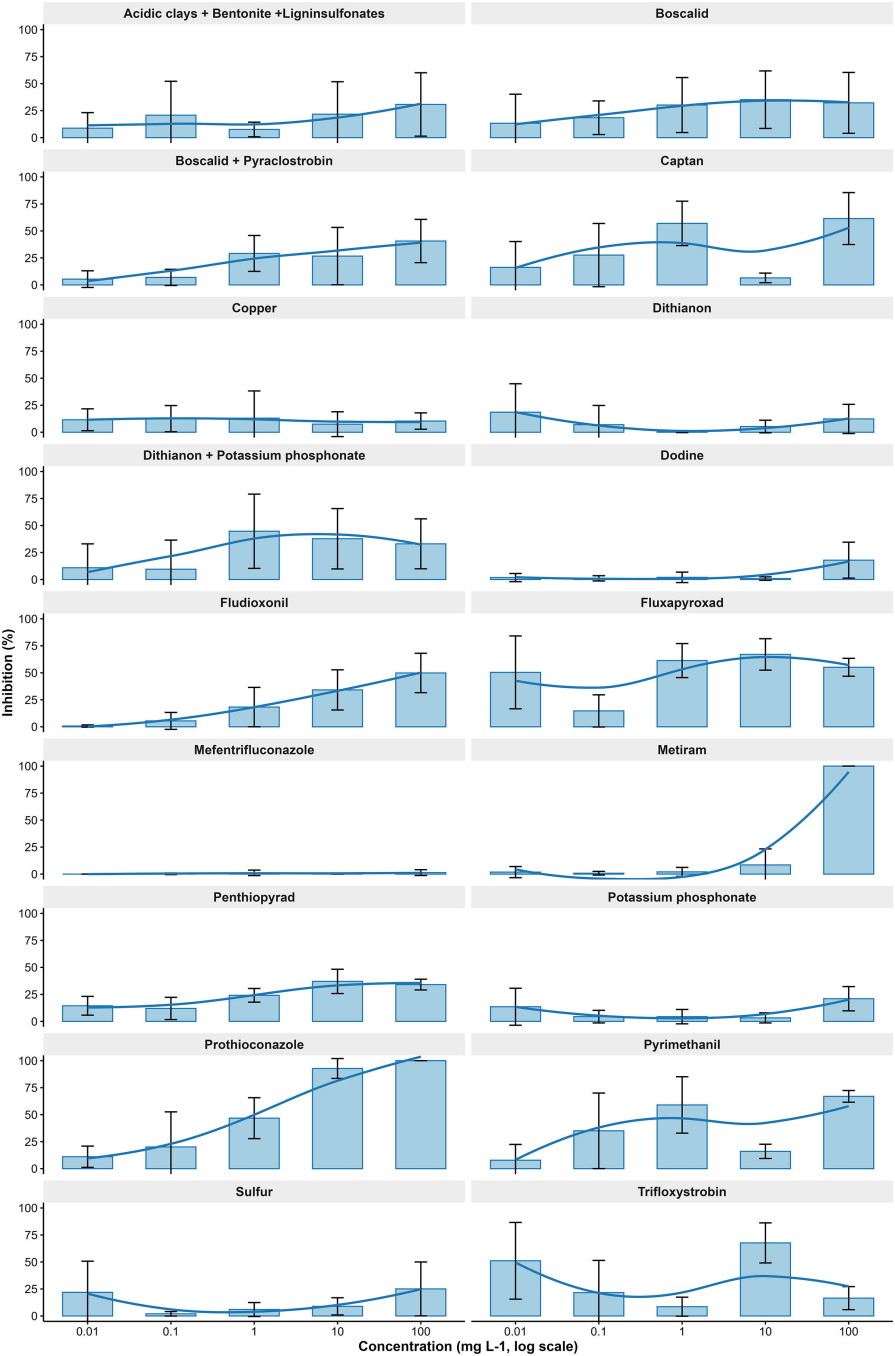
Inhibition profiles for each active substance (AS) across tested concentrations. Bars represent mean inhibition (%) ± standard deviation calculated from pooled data from two isolates, with four replicates per isolate and concentration (n = 8). The solid line shows a LOESS-smoothed curve fitted to the mean values to illustrate overall trends in inhibition. Concentrations are plotted on a logarithmic scale (mg L^−1^). Panels are separated by AS to facilitate comparison of inhibition patterns across treatments.

**Figure 2:**
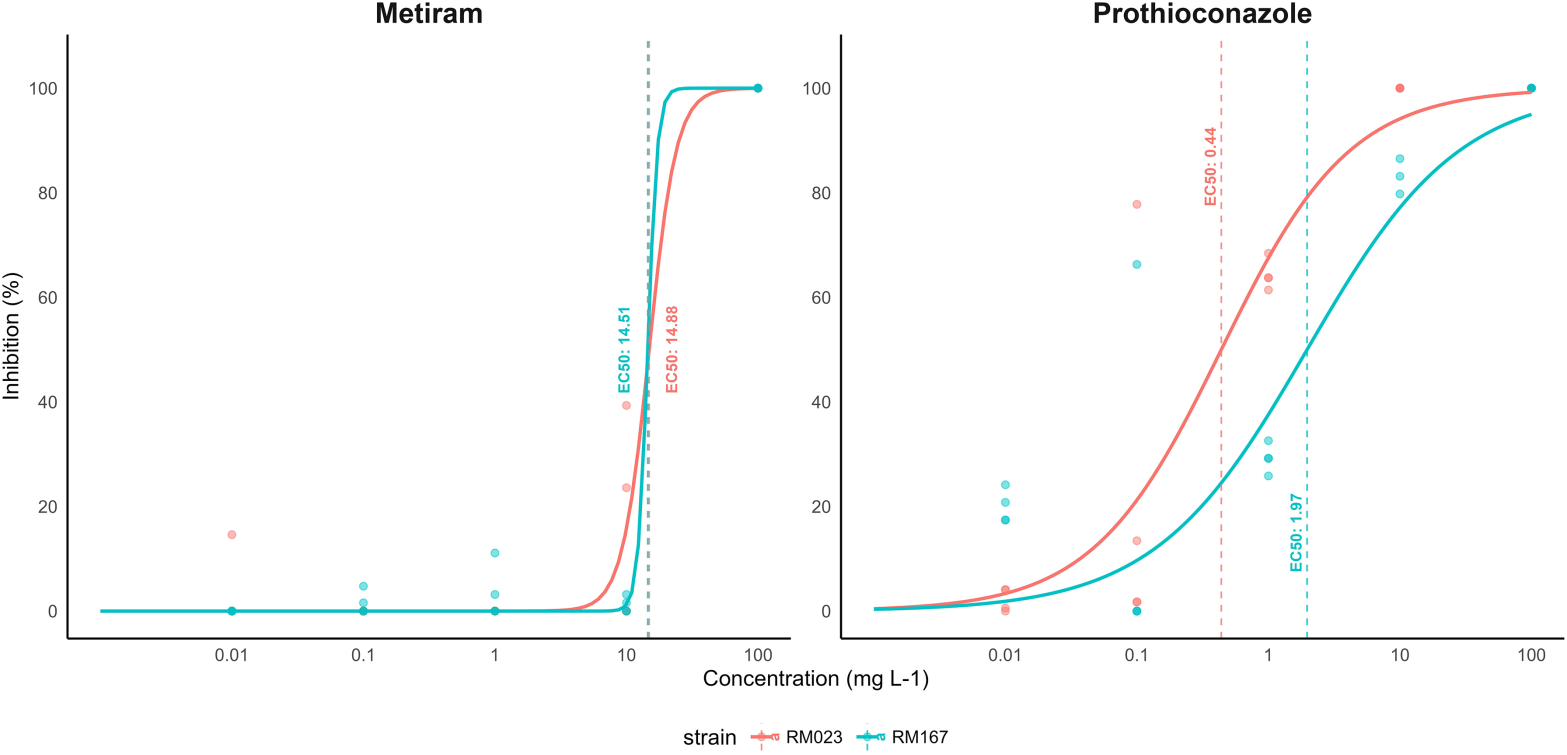
Dose–response curves and EC_50_ estimates for both isolates of the two active substances metiram and prothioconazole. Four-parameter log-logistic (LL.4) models were fitted over a logarithmic concentration range from the minimum tested concentration to 100 mg L^−1^. Points represent individual replicate measurements; four replicates were assessed for each isolate–active substance–concentration combination (n = 4). Smoothed model predictions are plotted alongside the individual inhibition data points, with vertical dashed lines indicating the estimated EC_50_ values. Labels summarize the numerical EC_50_ estimates for both isolates.

Mycelial growth inhibition of the *R. mali* isolates varied widely among the tested fungicides, and no FRAC group showed a consistently superior inhibitory effect across concentrations (**Figure 1**). At the highest tested concentration (100 mg L^−1^), fungicide treatments showed a range of inhibitory effects; however, pairwise comparisons indicated that many active substances were not significantly different from each other. Estimated marginal means revealed a gradual distribution of efficacy with substantial overlap among treatments. Prothioconazole exhibited the highest inhibition and formed a distinct group, differing significantly from most other active substances. Metiram also showed high inhibitory activity but was not significantly different from captan, which displayed an intermediate performance. Captan occupied an intermediate position and overlapped with both higher- and lower-performing fungicides. Most remaining active substances formed a large group with no significant differences among them at this concentration, indicating broadly comparable levels of inhibition. Overall, only a limited number of pairwise contrasts were statistically significant after correction for multiple testing, reflecting substantial overlap in efficacy among treatments at 100 mg L^−1^. Among the tested fungicides, only metiram and prothioconazole showed complete inhibition and a sigmoidal inhibition curve (**Figure 2**). Prothioconazole showed the lowest EC_50_ values for both isolates among the active substances for which dose–response curves could be fitted.

### 3.2 Field trials – fungicides and plant fortifier

**Figure 3:**
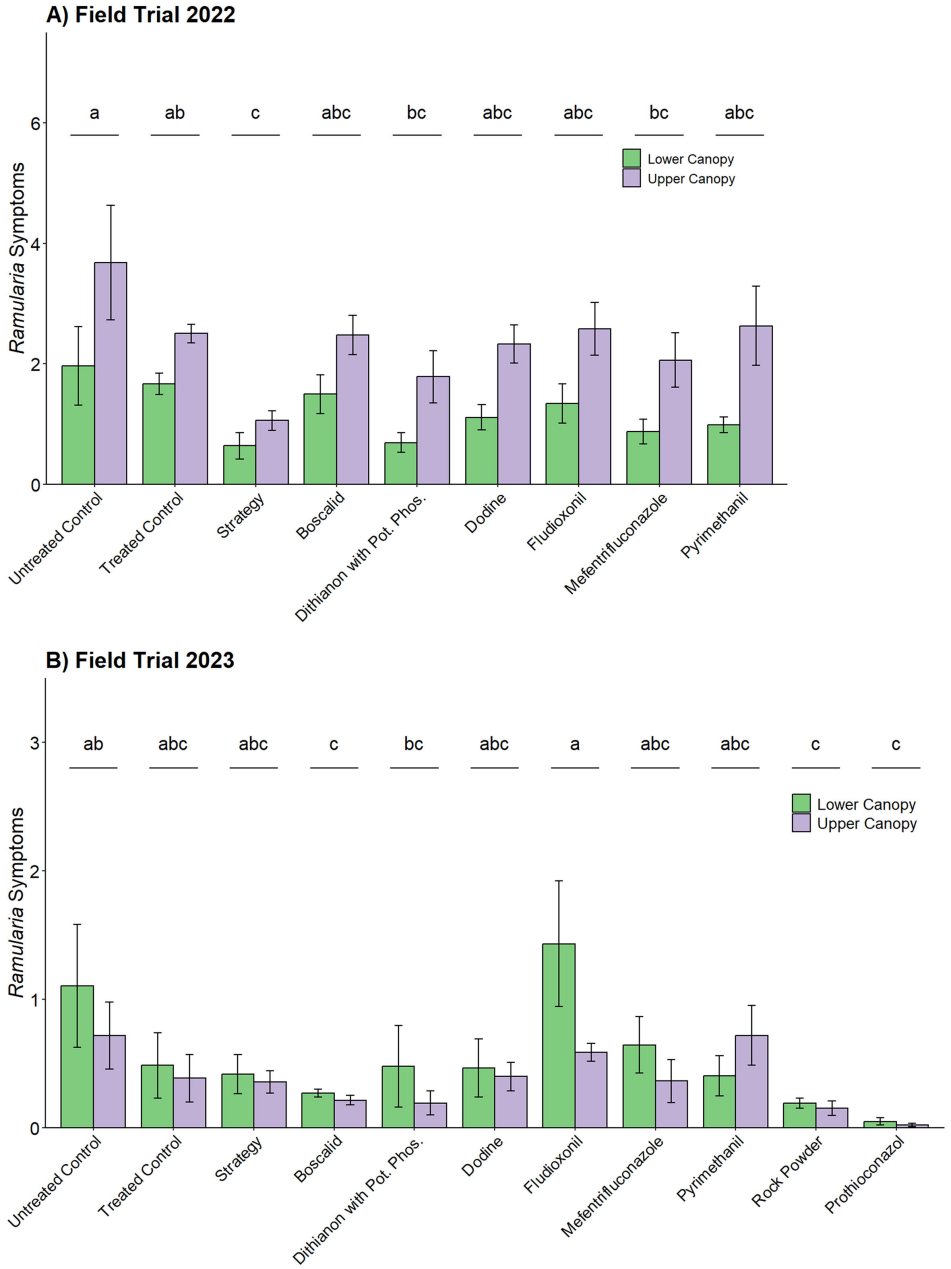
Mean area of dry lenticel rot in cm² (± SE) after different treatments (n = 5) in **(A)** 2022 and **(B)** 2023. In 2022, the strategy consisted of pyrimethanil and fludioxonil, boscalid, pyraclostrobin, and dithianon with potassium phosphonate, while in 2023 the strategy consisted of five treatments with dodine and a final treatment with fludioxonil. Means sharing the same letter are not significantly different (P ≤ 0.05).

In the field trial conducted in 2022, *Ramularia* infection on apple fruit was significantly affected by the tested treatments (ANOVA: F = 4.56; df = 8; P < 0.001) (**Figure 3A**). Apples from the untreated control exhibited the largest symptomatic areas, with mean infection values of 1.97 ± 0.65 cm² in the lower canopy and 3.68 ± 0.95 cm² in the upper canopy. In contrast, the fungicide strategy, dithianon with potassium phosphonate, and the mefentrifluconazole treatment led to a significant reduction in dry lenticel rot (Tukey HSD, P ≤ 0.05). The fungicide strategy (pyrimethanil and fludioxonil, boscalid, pyraclostrobin, and dithianon with potassium phosphonate) resulted in the lowest infection levels, reducing the mean symptomatic area to 0.64 ± 0.22 cm² in the lower canopy and 1.06 ± 0.16 cm² in the upper canopy, corresponding to an overall reduction of 69% compared with the untreated control and 60% compared with the treated control. Furthermore, dithianon in combination with potassium phosphonate reduced dry lenticel rot by 58% compared with the untreated control and by 44% compared with the treated control. Mefentrifluconazole reduced dry lenticel rot by 50% compared with the untreated control and by 32% compared with the treated control. Among all treatments in 2022, only the fungicide strategy achieved a statistically significant reduction when compared with the treated control (Tukey HSD, P ≤ 0.05). Across treatments, dry lenticel rot severity was higher in apples from the upper canopy than in apples from the lower canopy.

In 2023, treatment effects on dry lenticel rot were also significant (ANOVA: F = 4.25; df = 10; P < 0.001) (**Figure 3B**). Overall disease pressure was lower than in 2022, as indicated by reduced symptomatic areas in the untreated control, with mean infection values of 1.10 ± 0.48 cm² in the lower canopy and 0.72 ± 0.26 cm² in the upper canopy. Boscalid, prothioconazole, and acidic clay led to a significant reduction in symptomatic fruit surface area compared with the untreated control (Tukey HSD, P ≤ 0.05). The strongest reductions were observed for prothioconazole, followed by acidic clay and boscalid, with mean reductions of 96%, 81%, and 73%, respectively. In contrast to 2022, symptomatic areas were generally higher in fruit sampled from the lower canopy than from the upper canopy, indicating that canopy-related patterns were not consistent across years.

### 3.3 Field trials – 1-Methylcyclopropene

**Figure 4:**
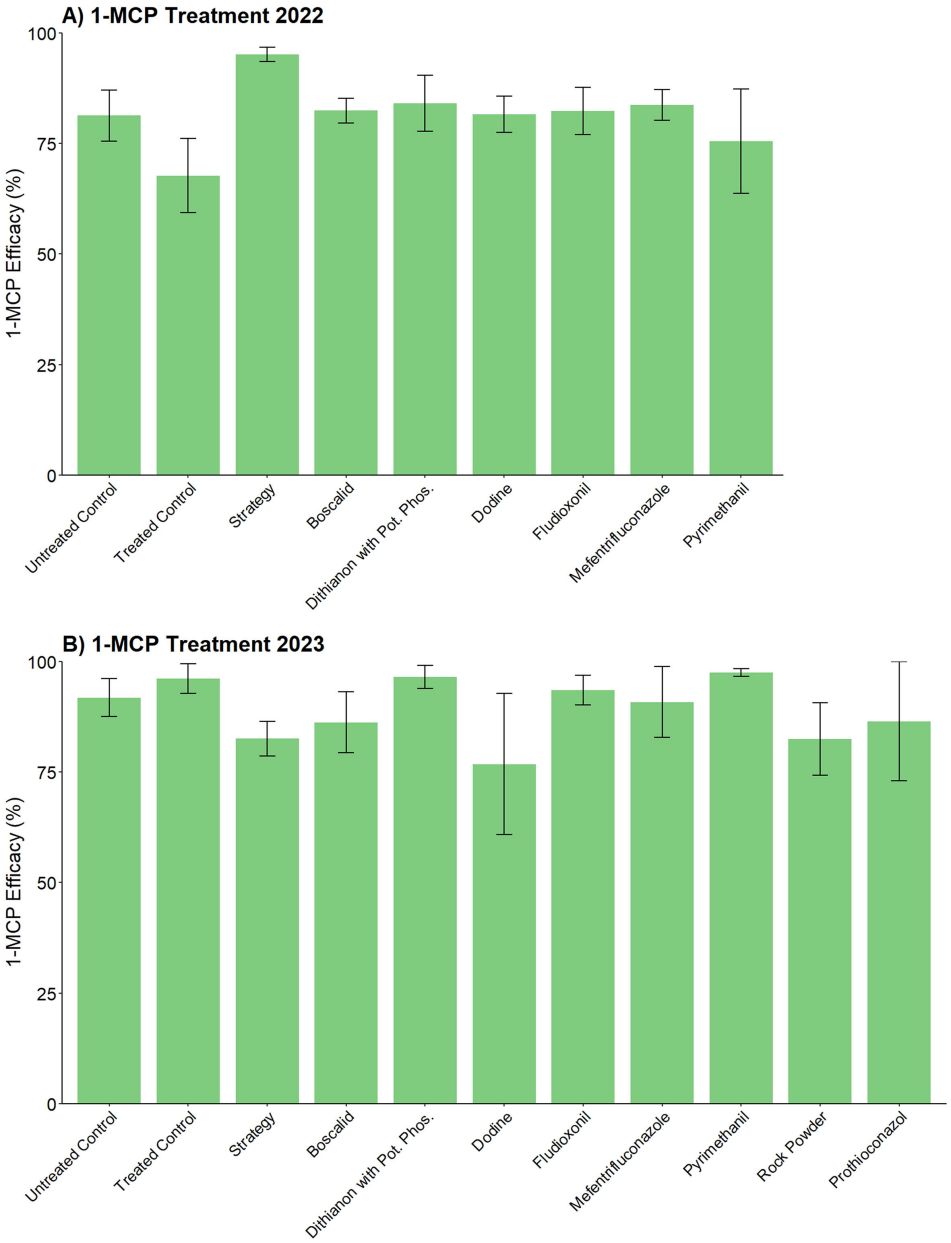
Mean reduction (%) in *Ramularia* symptomatic area by 1-methylcyclopropene (1-MCP), after different fungicide treatments in the field (n = 4) in **(A)** 2022 and **(B)** 2023. Means sharing the same letter are not significantly different (P ≤ 0.05).

Across both experimental years, postharvest treatment with 1-MCP reduced the expression of dry lenticel rot on stored apples, irrespective of the preharvest fungicide treatment applied (**Figure 4**). Across both years, 1-MCP efficacy ranged from 68% in the treated control in 2022 to 97% in the pyrimethanil treatment in 2023. Overall, postharvest application of 1-MCP resulted in an 82% reduction in dry lenticel rot symptoms in 2022 and an 89% reduction in 2023.

No significant interaction between fungicide treatments and 1-MCP efficacy was detected in 2022 (ANOVA: F = 1.36; df = 8; P = 0.25), indicating that differences in preharvest disease management did not influence the protective effect of 1-MCP during long-term storage. Similar results were obtained in the 2023 trial, confirming the absence of a significant influence of fungicide treatment on 1-MCP performance (ANOVA: F = 1.26; df = 10; P = 0.29).

Overall, these results show that 1-MCP treatment reduced the development of dry lenticel rot during controlled-atmosphere storage. Importantly, the efficacy of 1-MCP was stable and independent of the intensity or type of preharvest fungicide programme, highlighting its potential as a postharvest tool to reduce symptom expression under commercial storage conditions.

## 4 Discussion

Despite the increasingly significant economic impact of *R. mali*, the lack of established fungicide sensitivity profiles has historically made it difficult to determine whether field control failures stem from insufficient product efficacy, the emergence of resistant populations, or suboptimal application timing relative to the pathogen’s infection cycle. The present study addresses this knowledge gap, revealing pronounced variability in the *in vitro* sensitivity of the two tested *R. mali* isolates across diverse modes of action. Notably, no single fungicide class achieved consistent complete inhibition of mycelial growth *in vitro*. Both single-site and multi-site fungicides exhibited a broad range of efficacy, indicating that, for the isolates tested here, sensitivity of *R. mali* is strongly compound-specific rather than predictable from FRAC group assignment alone. These *in vitro* findings are further supported by field experiments conducted during the 2022 and 2023 growing seasons, which demonstrated generally limited disease control under practical conditions. However, these findings are restricted to the tested products and may differ for other formulations of the same active substances.

In the field trials, overall fungicide efficacy against *R. mali* was moderate to low, with no significant symptom reduction for most treatments. Notable exceptions were observed for acidic clay and prothioconazole, which consistently resulted in substantially lower symptom expression. Acidic clay treatments reduced symptom expression by 81%, while prothioconazole achieved a 96% reduction. For prothioconazole, these results are consistent with the corresponding *in vitro* data, where complete inhibition of mycelial growth was observed. In contrast, the activity observed for the closely related DMI mefentrifluconazole was not measurable. Together, these findings indicate that *R. mali* remains highly sensitive to specific azole compounds, while showing limited responsiveness to others targeting the same biochemical pathway. However, given that this study characterized only two isolates, these patterns must be interpreted with caution; a broader screening of populations is required to determine if this selective sensitivity is a stable trait across the species or subject to isolate-specific variation.

Multi-site fungicides displayed variable and often limited efficacy *in vitro*, a pattern that was largely reflected under field conditions. Although several multi-site compounds, namely copper, sulfur, metiram, and captan, showed minimal inhibition, metiram was the only active substance from this group to fully inhibit growth *in vitro*, yet it was omitted from the field trials due to the loss of authorization within the European Union in 2024.

In field trials, acidic clay consistently provided strong disease suppression compared with its performance *in vitro*. The mechanism of this registered plant fortifier is not fully understood, but these results suggest that the reduction of symptom expression may be driven by modification of the fruit surface, a physical barrier to infection, or hydrophobic effects of the compounds that cannot be captured by standard *in vitro* assays. However, the strong and consistent performance of acidic clay across both growing seasons highlights its potential value as a complementary disease management tool.

The integration of 1-MCP treatments further improved disease control. Across treatments, 1-MCP reduced symptom expression by 82% in 2022 and 89% in 2023, with individual treatment combinations reaching up to 97% efficacy. Because 1-MCP acts on ethylene-mediated ripening processes rather than directly on the pathogen, its effect is likely linked to delayed senescence or reduced susceptibility of the host tissue.

These results suggest that 1-MCP can provide an additional level of disease suppression when used after field treatments, even when individual fungicides show limited efficacy. The reduced performance of combinations involving fludioxonil and pyrimethanil aligns with their moderate *in vitro* activity and suggests limited complementary effects with 1-MCP under field conditions.

The most pronounced disease suppression was observed with the combination of 1-MCP treatment and prothioconazole, resulting in *R. mali* control up to 100%. This outcome underscores the potential of integrating highly effective single-site fungicides with pre-storage treatments to achieve robust disease control.

However, from a resistance-management perspective, the combined *in vitro* and field data highlight both risks and opportunities. The strong and consistent efficacy of prothioconazole against the tested isolates suggests high sensitivity to this DMI in the tested material, but broader population-level screening is required before conclusions can be drawn about the sensitivity status of *R. mali* populations. Yet, reliance on a single highly effective azole may impose selection pressure on the CYP51 target and lead to shifting-type resistance (Brent and Hollomon, 2007; Rehfus et al., 2019). The contrasting response to mefentrifluconazole demonstrates that reduced efficacy to one DMI does not necessarily imply class-wide resistance, but it also illustrates how rapidly efficacy can differ among closely related compounds.

Furthermore, the alpine regions represent some of the most intensive apple-growing areas in Europe, where fungicide applications are primarily driven by the management of apple scab (*Venturia inaequalis*) and further major preharvest diseases such as powdery mildew (*Podosphaera leucotricha*), Alternaria leaf blotches and fruit spots (*Alternaria alternata* spp.), and Glomerella leaf spot (GLS; caused by *Colletotrichum* spp.). This creates a scenario of unintended selection pressure. The moderate performance of several single-site fungicides may reflect limited intrinsic sensitivity, previous selection pressure, or isolate-specific variation; broader population-level surveys are required to distinguish among these possibilities. The generally moderate performance of SDHIs and other single-site fungicides further emphasizes the need to avoid repeated use of any single mode of action (Brent and Hollomon, 2007). The pronounced benefits observed from combining fungicides with 1-MCP treatments and acidic clays support integrated disease management strategies that diversify selection pressures. Such approaches, combining effective single-site fungicides with multi-site or non-chemical measures, are likely to be critical for sustaining long-term control of *R. mali* and mitigating the development of fungicide resistance.

In conclusion, this study provides a comparative assessment of *in vitro* fungicide sensitivity in two *R. mali* isolates, alongside field efficacy and postharvest 1-MCP treatment against dry lenticel rot. Because the *in vitro* sensitivity assay was limited to two isolates, these results should be regarded as an initial screening rather than a population-level characterization of fungicide sensitivity in *R. mali*, and broader isolate collections spanning multiple orchards and regions are needed before conclusions can be drawn about baseline sensitivity at the population level. Most tested fungicides provided limited control in the field, whereas prothioconazole and acidic clay showed the strongest field performance among the evaluated preharvest treatments. Postharvest application of 1-MCP consistently reduced symptom expression during controlled-atmosphere storage; however, the underlying mechanism remains unresolved. Because physiological fruit-quality parameters confirming ripening status or senescence progression were not assessed, a direct relationship between disease expression and fruit senescence cannot be concluded from the present data. For commercial growers, the results indicate that dry lenticel rot is unlikely to be managed reliably by standard fungicide programmes alone. Instead, management should combine targeted preharvest measures with postharvest approaches that reduce symptom development during storage, including 1-MCP treatment where its use is permitted and compatible with storage and marketing requirements. Acidic clay may provide an additional complementary tool for integrated or reduced-residue production systems, but further validation across cultivars, orchards, and environmental conditions is required. Future studies should include broader isolate collections representative of the pathogen population, additional growing regions, and physiological fruit-quality parameters to clarify whether the effect of 1-MCP is linked to delayed ripening, altered host susceptibility, or other storage-related mechanisms.

## Data Availability

The raw data supporting the conclusions of this article will be made available by the authors, without undue reservation.
